# Potential Use of *Neisseria meningitidis* Serogroup B Vaccines to Prevent *Neisseria gonorrhoeae* Infection: Epidemiological Considerations

**DOI:** 10.1002/jia2.70155

**Published:** 2026-07-24

**Authors:** Sami L. Gottlieb, Jane Rowley, Nuria Balibrea, Dominique A. Caugant, Ray Borrow, Antoine Durupt, Helen S. Marshall, Maeve B. Mello, Marco Aurelio Sáfadi, Marie‐Pierre Preziosi

**Affiliations:** ^1^ World Health Organization Geneva Switzerland; ^2^ Norwegian Institute of Public Health Oslo Norway; ^3^ UK Health Security Agency, Manchester Royal Infirmary Manchester UK; ^4^ Adelaide University and the Women's and Children's Health Network Adelaide South Australia Australia; ^5^ Santa Casa de São Paulo School of Medical Sciences São Paulo Brazil

**Keywords:** epidemiology, *Neisseria gonorrhoeae*, *Neisseria meningitidis*, vaccination

## Abstract

**Introduction:**

Clinical trials are evaluating the efficacy of serogroup B meningococcal (MenB) outer membrane vesicle (OMV) vaccines in preventing gonorrhoea. We assessed the global epidemiology of gonorrhoea and MenB invasive meningococcal disease (IMD) and reviewed national MenB‐OMV vaccination policies to inform potential use of these vaccines.

**Methods:**

We included country‐specific epidemiologic data from 2015 through October 2025. Gonorrhoea prevalence data for general populations of women were extracted from published systematic reviews and case reports taken from well‐established reporting systems. Country‐specific MenB IMD incidence rates were drawn from published reviews and surveillance reports. National MenB‐OMV immunization policies were obtained from published reviews and databases from the World Health Organization and vaccine manufacturers.

**Results:**

Forty‐two countries had ≥1 gonorrhoea prevalence study. Mean prevalence was <1.0% in 19 (45%) countries, 1.0%−2.49% in 10 (24%), 2.5%−4.99% in 10 (24%) and ≥5% in three (7%), with higher prevalences mostly in the WHO African Region. MenB IMD incidence was reported in 62 countries: 17 (27%), mainly in the WHO African Region, had no cases; 28 (45%) had incidence <0.25/100,000; 14 (23%) had incidence 0.25−0.49/100,000; and only three had incidence ≥0.5/100,000 annually. Only 15 countries had data for both conditions. Case‐report data showed gonorrhoea incidence peaking at ages 20–24 and being substantially higher among men who have sex with men (MSM). MenB IMD incidence was highest among children <4 years, with a second peak among 15‐ to 24‐year‐olds in some countries. Twenty‐one countries incorporated MenB‐OMV vaccines into infant immunization programmes and seven into adolescent programmes. One country had a targeted MenB‐OMV vaccination programme (MSM at high‐risk) for gonorrhoea prevention.

**Discussion:**

Epidemiologic data were lacking in many countries; only a handful had a substantial burden of both gonorrhoea and MenB IMD. Low‐ and middle‐income countries with high gonorrhoea prevalence typically reported no MenB IMD or lacked data, whereas high‐income countries with higher MenB IMD incidence and MenB‐OMV vaccine use had low gonorrhoea prevalence but high rates in subpopulations like MSM.

**Conclusions:**

If trials confirm MenB‐OMV vaccine cross‐protection against gonorrhoea, global epidemiology can help identify settings and populations for potential vaccine use against gonococcal infection alone, or for both conditions. Improved data collection and cost‐effectiveness analyses across both conditions can further inform decision‐making.

## 1 Introduction

*Neisseria gonorrhoeae* (gonococcus) is an obligate human bacterial pathogen causing the sexually transmitted infection (STI) gonorrhoea. In 2020, an estimated 82 million new gonococcal infections occurred in people aged 15−49 years globally [1]. A disproportionate share of these infections occur in low‐ and middle‐income countries (LMICs), where resources for STI diagnosis and treatment are limited [1]. If left untreated, gonococcal infection can lead to several adverse sexual and reproductive health outcomes, including pelvic inflammatory disease, ectopic pregnancy, infertility, adverse pregnancy outcomes and neonatal conjunctivitis. Gonococcal infection has also been linked to an increased risk of HIV acquisition and transmission [2].

The rapid emergence of *N. gonorrhoeae* strains with reduced susceptibility or resistance to multiple classes of antibiotics, including extended‐spectrum cephalosporins, along with the health consequences of untreated gonorrhoea, has made reducing the incidence of *N. gonorrhoeae* infection a global public health priority [3, 4]. Scaling up existing prevention and treatment services is essential. However, given challenges in implementing existing interventions [5] along with increasing gonococcal antimicrobial resistance (AMR) [3], this will likely not be sufficient for sustainable control. New prevention tools are needed, and developing vaccines against *N. gonorrhoeae* infections is a World Health Organization (WHO) global STI research priority [6]. No licensed gonococcal vaccines exist. One gonococcus‐specific vaccine candidate (*Neisseria gonorrhoeae* generalized modules for membrane antigens vaccine, GSK) was recently in early clinical trials (NCT05630859). However, as of April 2026, no trial results have been released, but the developer has indicated it will not pursue further development [7]. Although several promising candidates are in pre‐clinical stages, it may be many years before an efficacious gonococcal‐specific vaccine is available [8].

At the same time, observational data suggest that existing vaccines against a related pathogen, serogroup B *Neisseria meningitidis* (MenB), may provide cross‐protection against *N. gonorrhoeae*. *N. meningitidis* (meningococcus) is a bacterium sharing 80%–90% genetic homology with *N. gonorrhoeae* [9]. Primarily transmitted through respiratory and oropharyngeal secretions, *N. meningitidis* is often associated with asymptomatic carriage in the nasopharynx. In rare circumstances, it can cause invasive meningococcal disease (IMD), a life‐threatening acute infection most commonly involving meningitis or sepsis [10]. MenB is one of the six most common pathogenic serogroups of *N. meningitidis*, which can cause endemic disease or seasonal outbreaks. Unlike serogroups A, C, W, Y and X, for which conjugate vaccines are available, MenB disease prevention relies on novel recombinant protein‐based vaccines.

In a few countries with outbreaks due to MenB, mass MenB vaccination campaigns were associated with declines in reported cases of gonorrhoea [11, 12]. These countries were all using tailor‐made strain‐specific MenB vaccines based on outer membrane vesicles (OMVs) for antigen presentation, where Porin A is the immunodominant antigen. Since then, multiple observational studies and one small randomized controlled trial (RCT) have evaluated this potential cross‐protection by OMV‐based MenB vaccines, with meta‐analyses estimating pooled vaccine effectiveness of 30%−35% in preventing gonococcal infection [11, 13, 14].

Two MenB vaccines are commercially available to prevent MenB IMD: 4CMenB (Bexsero, GSK) and MenB‐FHbp (Trumenba, Pfizer). 4CMenB contains an OMV based on a New Zealand MenB strain plus three additional outer membrane proteins: *Neisseria* adhesin A (NadA), factor H binding protein (fHbp) and Neisserial Heparin Binding Antigen (NHBA). MenB‐FHbp contains two variants of fHbp but does not contain an OMV component and does not show evidence of cross‐protection against gonorrhoea [15]. After vaccination with 4CMenB, antibodies recognize gonococcal proteins, providing biological support for cross‐protection [16, 17]. Additionally, 4CMenB has been shown to reduce oropharyngeal carriage of unencapsulated, but not encapsulated, meningococci; since gonococci are unencapsulated, cross‐reactive antibodies may bind more easily due to increased exposure of outer membrane proteins [18].

As of December 2025, six RCTs assessing the efficacy of 4CMenB in preventing *N. gonorrhoeae* infection are ongoing: three among men who have sex with men (MSM); a multi‐centre RCT among adult men and women at higher risk of gonococcal acquisition in the United States, Thailand and Malawi; an RCT among cis‐gender women in Southern Africa; and an efficacy assessment in a controlled human infection model (Table 1). Results from most of the RCTs should be available by the end of 2026. If these trials demonstrate some efficacy against gonococcal infection, the public health community needs to consider how MenB OMV vaccines might best be used in different contexts.

**TABLE 1:** Randomized controlled trials of the efficacy of *Neisseria meningitidis* serogroup B vaccines for preventing gonococcal infection, as of December 2025.

Trial number	Phase or type of study	Study name	Location, numbers	Population	Results expected
ACTRN12619001478101	Phase 3 RCT efficacy, immunogenicity	MenGO: Does the licensed meningococcal vaccine Bexsero provide cross‐protection against gonorrhoea?	Australia 130	Men who have sex with men (MSM)	Early 2026
NCT04415424	Phase 3 RCT efficacy, immunogenicity	GoGoVax: Efficacy study of 4CMenB (Bexsero) to prevent gonorrhoea infection in gay and bisexual men	Australia 730	MSM	Early 2026
NCT05766904	RCT efficacy	Efficacy trial on meningococcal B vaccine for preventing gonorrhea infections	Hong Kong 150	MSM	Early 2026
NCT04350138	Phase 2 RCT efficacy	Safety and efficacy study of meningococcal group B vaccine rMenB+OMV NZ (Bexsero) to prevent gonococcal infection	USA, Thailand, Malawi 2200	Adults at higher risk of gonorrhoea	Mid‐2026
NCT06446752	Phase 3 RCT efficacy	BIYELA: Efficacy of Bexsero in preventing gonococcal infection among South African cis‐gender women	South Africa 1100	Cis‐gender women	Late 2026
NCT05294588	RCT efficacy in a controlled human infection challenge model	Efficacy of immunization with 4C‐MenB in preventing experimental urethral infection with *Neisseria gonorrhoeae*	USA 140	Men	2028

Abbreviations: MSM, Men who have sex with men; RCT, randomized controlled trial.

In 2021, WHO published preferred product characteristics (PPCs) for gonococcal vaccines, based on expert consultations on the potential value and optimal use of these vaccines to meet the global public health need [19, 20]. Experts highlighted the need for further information on how gonococcal infection epidemiology overlaps with that of MenB disease to inform possible future use of MenB vaccines for both pathogens. In this article, we present a scoping review of the global epidemiology of gonococcal infection and of MenB disease, describe the overlap between the two conditions and explore the relationship with national MenB vaccination policies to highlight important considerations for potential future use of MenB vaccines to prevent gonococcal infection.

## 2 Methods

We conducted a scoping review of global gonococcal and meningococcal epidemiology and MenB vaccine use, using the most recent systematic reviews and other comprehensive data sources. We examined epidemiologic overlap between the two conditions across and within countries, along with MenB vaccination policies. Ethics Board review and consent were not required, as all data were from previously published studies or publicly available assessments.

### 2.1 Epidemiology of *Neisseria gonorrhoeae* Infection

Our estimates of the global distribution of gonococcal infection were based on prevalence studies among general populations of women. We focused on prevalence rather than case‐reporting data, given the large proportion of asymptomatic infections, and focused on women, given limited data for men across countries outside of symptomatic or high‐risk populations.

We based our scoping review of gonococcal infection epidemiology on 13 multi‐country systematic reviews published in 2020 or later, through 1 October 2025 [21, 22, 23, 24, 25, 26, 27, 28, 29, 30, 31, 32, 33]. The reviews were used to identify primary studies reporting on gonorrhoea prevalence in general populations of sexually active women of reproductive age, which included pregnant women, women attending family planning clinics, female students and women attending primary healthcare clinics for non‐STI‐related visits. The reviews were supplemented by a PubMed and Embase search on “gonorrhoea” and “prevalence” to identify additional studies published in 2024 or 2025 (latest search 1 October 2025).

We included studies meeting the following criteria: (1) used nucleic acid amplification tests on urine, cervical or vaginal specimens; (2) had a sample size of ≥100; and (3) the midpoint of specimen collection was in 2015 or later (for studies without these dates, specimen collection was assumed to be 2 years before the publication date). For countries with multiple data points, we calculated a weighted mean prevalence. Countries were allocated into one of six categories of gonorrhoea prevalence: <0.5%, 0.5%−0.99%, 1%−2.49%, 2.5%−4.99%, ≥5% or no data.

The distribution of gonococcal infections within countries by age, sex, risk group and over time were based on incident case reports from four settings with well‐developed reporting systems for gonorrhoea (the United States, the United Kingdom, the European Union and Australia) [34, 35, 36, 37].

### 2.2 Epidemiology of *Neisseria meningitidis* Serogroup B Disease

The scoping review of global MenB IMD epidemiology was based on a series of global and regional assessments of available country‐specific IMD surveillance data conducted since 2017 by the Global Meningococcal Initiative [38, 39, 40, 41, 42, 43, 44]. These were supplemented with surveillance data from 2015 onwards from the European Centre for Disease Prevention and Control Disease Atlas [45], the Pan American Health Organization [46] and the WHO African Region enhanced surveillance of meningitis [47]. All sources were searched through 1 October 2025. We also evaluated the most recent global literature reviews of individual published studies of MenB [48, 49] and IMD epidemiology [50, 51] for any studies from 2015 or later.

Country‐specific MenB incidence rates per 100,000 population from 2015 or later were extracted from these sources. If countries had multiple years of data since 2015, we used the latest year available. Estimates spanning multiple years were extracted if they included data from 2015 or later. For countries that did not have MenB‐specific rates but had estimates of overall IMD incidence and the proportion of isolates that were MenB, we multiplied these two figures to estimate MenB incidence rates. Countries with only overall IMD rates without the proportion that were MenB, or which had numbers of MenB cases without any indication of the underlying denominator, were not included. Countries were allocated into one of five categories: (1) no MenB IMD reported; (2) incidence <0.25/100,000; (3) incidence 0.25−0.49/100,000; (4) incidence ≥0.5/100,000; (5) no data or data insufficient to calculate MenB incidence. Only countries with enhanced surveillance, serotyping of isolates and documented “zero” MenB cases were included in the “no MenB” category.

Where available, age‐ and population‐specific incidence rates of MenB disease from the global and regional surveillance assessments and the additional published review articles were used to evaluate the distribution of MenB IMD within countries. Information about outbreaks or trends was also noted where available.

### 2.3 Use of *Neisseria meningitidis* Serogroup B OMV Vaccines

The primary sources of data on MenB vaccine policies by country were two reviews of 4CMenB licensure/registration and policies published in 2022 [52, 53], supplemented by national vaccine schedule data collected through the WHO/UNICEF Joint Reporting Form through the end of 2024 [54]. We also contacted GSK in November 2025 for any new country registration and policies for 4CMenB tracked through their databases. Countries were categorized into four groups: (1) vaccine not registered/licensed; (2) vaccine registered/licensed but no national recommendations; (3) vaccine recommended for use in one or more populations; (4) vaccine incorporated into National or Regional Immunization Programme.

## 3 Results

### 3.1 Global Distribution of Gonococcal Infection and Serogroup B Meningococcal Disease

**FIGURE 1:**
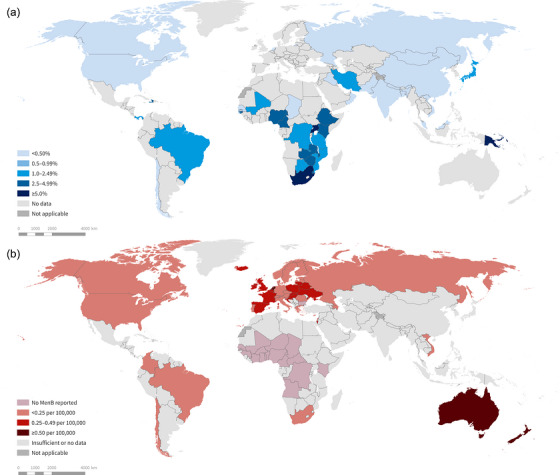
Global distribution of gonococcal infection prevalence and serogroup B invasive meningococcal disease incidence, data from 2015 or later. Note different scales. (a) Prevalence (%) of *N. gonorrhoeae* in general populations of sexually active women, by country. (b) Yearly incidence per 100,000 of serogroup B invasive meningococcal disease, by country. MenB, *Neisseria meningitidis* serogroup B.

For gonorrhoea, the review identified 113 data points from 42 countries meeting study entry criteria; 21 countries had more than one data point. Figure 1A shows estimated gonorrhoea prevalence values in the general population of sexually active women of reproductive age, by country. In the 42 countries with data, estimated gonorrhoea prevalence was ≥5% in three countries (7.1%), between 2.5% and 4.99% in 10 countries (23.8%), between 1% and 2.49% in 10 countries (23.8%) and <1% in 19 countries (45.2%) (Figure 1A). Eleven of the 13 countries with a prevalence ≥2.5% were in sub‐Saharan Africa. For many high‐income countries, particularly in Europe, no data were available because the most recent general‐population prevalence studies, typically with very low gonorrhoea prevalence, were conducted before 2015.

For MenB IMD, data to estimate incidence were available for 62 countries (Figure 1B). The WHO European Region had the most countries with data (*n* = 36). Incidence rates ranged from 0.00 to 0.84 per 100,000 population. Of the 62 countries, 17 (27.4%), all but one in the WHO African Region, had no MenB IMD cases; 28 (45.2%) had MenB cases but a yearly MenB IMD incidence <0.25 per 100,000; 14 (22.6%) had incidence between 0.25 and 0.499 per 100,000; and only three countries (Australia, the Netherlands and New Zealand) reported an annual incidence rate ≥0.5 per 100,000. All countries with rates ≥0.25 per 100,000 were from the WHO European Region or Australasia.

Only 15 countries in our review had data meeting the inclusion criteria for both gonorrhoea and MenB IMD. Seven of these countries had gonorrhoea prevalence ≥1%, of which five (all in Africa) had no MenB and two (Brazil and South Africa) had low MenB IMD incidence (<0.25 per 100,000). The remaining eight countries all had gonorrhoea prevalence <0.49%; all but one of these (the Netherlands) had low MenB IMD incidence.

### 3.2 Distribution of Gonorrhoea Cases and MenB Disease Within Countries

**FIGURE 2:**
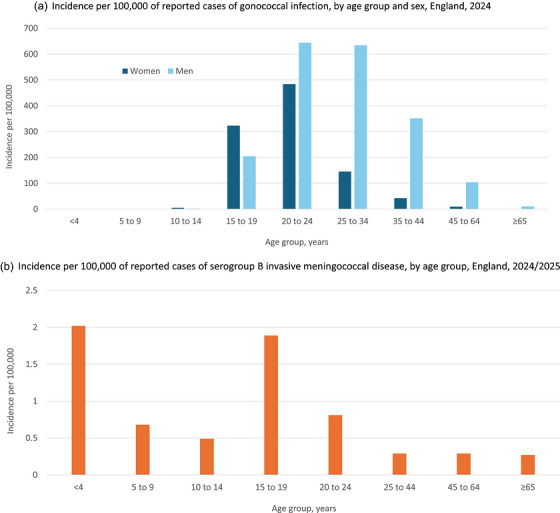
Age distribution of gonococcal infection and serogroup B meningococcal disease incidence in England. Data from [35, 56, 57]. Note different scales. (a) Incidence per 100,000 of reported cases of gonococcal infection, by age group and sex, England, 2024. (b) Incidence per 100,000 of reported cases of serogroup B invasive meningococcal disease, by age group, England, 2024/2025.

Within countries, considerable variation exists for both conditions by age, at‐risk groups and over time. For gonorrhoea, in countries with strong case reporting systems, the highest incidence of reported cases typically occurs among women in the 20‐ to 24‐year‐old age group, followed by the 15‐ to 19‐year‐old age group; for men, rates are highest in either the 20‐ to 24‐year‐old or 25‐ to 29‐year‐old age groups, followed by the 30‐ to 39‐year‐old age group. An illustrative age distribution for England is found in Figure 2A.

MenB IMD can occur at any age; however, in most settings, incidence is highest in infants and young children. Some countries, particularly high‐income settings, have observed a second peak in 15‐ to 24‐year‐olds [50, 51]. An illustrative age distribution, for England, is shown in Figure 2B. However, this apparent second peak in adolescence does not occur in all settings, for example this has not been recently observed in countries in Latin America [55].

For gonorrhoea, epidemiology varied substantially by risk group, most notably among MSM. In a global systematic review incorporating 188 study estimates from almost 350,000 MSM, the overall pooled prevalence of gonorrhoea was 7.2%, which was approximately 10 times greater than in the general population of men [58]. In 2023, MSM accounted for more than half (58%) of the reported cases in the 17 European countries collecting mode of transmission [36]. In Australia, active surveillance in sexual health clinics estimated an incidence of 36.4/100 person‐years among MSM living with HIV and 25.7/100 person‐years among HIV‐negative MSM [37]. Gonorrhoea prevalence among female sex workers is often many times higher than that among general populations of women, in studies with data from both populations [32]. Higher prevalence and incidence have also been observed among specific racial, ethnic or other marginalized minority populations with historical barriers to healthcare access.

Although individuals with certain health conditions, such as asplenia or complement disorders, and laboratory workers exposed to *N. meningitidis* are at higher risk of MenB IMD, these groups are a small proportion of the population. MenB can also cause outbreaks of IMD, generally linked to those living in close quarters (e.g. university students or military personnel) or to mass gatherings, but such outbreaks are usually sporadic, limited and unpredictable [49].

Available data on trends show that reported gonorrhoea cases have generally been increasing over the past decade among MSM, and stable or increasing in other populations, but in some settings have levelled off or even decreased in the past 2–3 years, with notable decreases during the COVID‐19 pandemic. In Australia, case rates more than doubled between 2014 and 2023 [37]. In the United States, gonorrhoea case reports per 100,000 population increased 110% among men (from 119.1 to 249.7) and 77% among women (from 100.4 to 177.9) between 2014 and 2021, and then declined to rates of 228.3 among men and 130.7 among women in 2023 [34].

Surveillance data indicate that the overall incidence of IMD for all serogroups has been falling over time globally, with further reductions during the COVID‐19 pandemic. However, following relaxation of COVID‐19 containment measures, increases in IMD cases have been observed in several regions, primarily driven by Men B. IMD cases caused by other serogroups have remained low in countries with well‐established meningococcal ACWY vaccination programmes. Thus, MenB is now the predominant serogroup causing IMD across Europe, North and South America, and Australasia [46, 50]. Nonetheless, the epidemiology of MenB disease tends to be dynamic, with substantial year‐to‐year variability and an ongoing risk of sporadic outbreaks.

### 3.3 Meningococcal B OMV‐Based Vaccine Use

**FIGURE 3:**
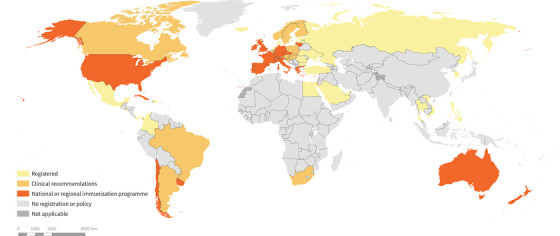
Status of outer membrane vesicle‐based meningococcal serogroup B vaccine licensure, recommendations and implementation, November 2025.

The MenB OMV vaccine 4CMenB was first licensed in 2013, and as of November 2025, 59 countries have licensed this vaccine ([52, 53, 54] and personal communication, GSK, see File S1), and one country (Cuba) has used a locally developed strain‐specific OMV vaccine since 1991 [48, 54] (Figure 3). To date, 20 countries have incorporated 4CMenB into their national or regional infant immunization programmes, and six of these also have programmes for adolescents. Cuba also has a national infant programme. One country (the United States) has a programme for adolescents and young adults aged 16–23 years based on shared clinical decision‐making [59]. Some countries, for example Canada, only implement routine 4CMenB use for outbreak control. An additional 16 countries have clinical recommendations for its use, primarily for infants or specific groups with at‐risk health conditions or exposures.

In addition, at least one country (the UK, as of August 2025) [60] and some other jurisdictions [61] have begun implementing 4CMenB vaccination for prevention of gonococcal infection, based on existing observational data. The UK programme targets MSM attending sexual health clinics with a high risk of acquiring gonococcal infection [60].

## 4 Discussion

Vaccines against *N. gonorrhoeae* infection are urgently needed because of increasing gonococcal AMR and the negative impact of untreated gonococcal infections on sexual and reproductive health. Observational data suggest that vaccines providing some protection against *N. gonorrhoeae* may already exist, in the form of MenB OMV vaccines. Several RCTs have been designed to directly assess the efficacy of the commercially available OMV vaccine 4CMenB in preventing *N. gonorrhoeae* infection, and most results will be available by the end of 2026. If these trials demonstrate some efficacy against gonococcal infection, whether and how these vaccines might be used to optimally and efficiently prevent both gonorrhoea and MenB IMD depend on an interplay of several factors. These factors include the public health need for the vaccine in different settings for each pathogen, the preferred target populations and delivery strategies for each, specific vaccine characteristics (e.g. efficacy and duration of protection) and policy factors such as cost‐effectiveness of the vaccine considering both conditions [20, 62]. In this scoping review, we evaluated the extent of the overlap in the global epidemiology of gonococcal infection and MenB disease, and the relationship with current MenB vaccine use across countries, to highlight important considerations for potential future use of MenB OMV vaccines for gonococcal infection in addition to MenB disease.

We found that for many countries, data are lacking for both gonococcal infection and invasive MenB disease. This lack of information is a challenge in understanding their overlap; however, existing data reveal several key patterns. Many of the highest burden areas for gonococcal infection, primarily LMICs with a historical lack of access to affordable STI testing and treatment programmes, have little known MenB disease or no data. Multiple countries conducting enhanced meningitis surveillance in the African meningitis belt had a near‐absent incidence of MenB disease reported, despite consistent detection of other serogroups. The reasons for this are not fully understood, but it has been postulated to reflect a combination of environmental, immunological and meningococcal strain factors that preferentially facilitate transmission of other serogroups and limit establishment of MenB lineages [44, 63]. Conversely, most of the countries with a substantial history of endemic MenB disease or MenB outbreaks, and which are using OMV‐based MenB vaccines, are high‐ or upper‐middle‐income countries with low gonococcal infection prevalence in the general population. Although gonorrhoea prevalence data for general populations were unavailable for many countries with the highest MenB IMD rates—such as those in Europe and Australasia—this largely reflects the fact that overall gonorrhoea prevalence had been sufficiently low that general‐population prevalence studies were no longer carried out after 2015. For example, a national UK survey from 2010 to 2012 estimated gonorrhoea prevalence in 16‐ to 44‐year‐olds of <0.1% [64]. However, case‐reporting data from these same settings make clear that despite low general‐population prevalence, gonorrhoea rates are high in specific sub‐populations, particularly MSM. In the few countries with both endemic MenB IMD and gonococcal infection data, the prevalence of gonorrhoea in the general population and the incidence of MenB disease were typically low (<0.5% and <0.25/100,000, respectively), with a few exceptions (e.g. Brazil and South Africa).

In countries with high general‐population gonorrhoea prevalence, such as Papua New Guinea, South Africa and Uganda, broad use of MenB OMV vaccines might be valuable for preventing gonococcal infection alone, regardless of MenB IMD. The degree of benefit would depend on demonstrated efficacy in the RCTs, along with cost‐effectiveness, feasibility and overall programmatic costs. Mathematical models have predicted that gonococcal vaccines with efficacy as low as 20%–30%, similar to what has been observed in the observational studies of MenB OMV vaccines and gonorrhoea [11, 13, 14], could potentially have a substantial effect on gonococcal infection prevalence at a population level [65]. Vaccines with this efficacy have also been predicted to be cost‐effective (or even cost‐saving) and to potentially reduce AMR in some settings [65, 66, 67]. However, the level of impact depends on the extent of vaccine uptake in populations at risk and the duration of protection. In countries with lower general‐population prevalence, use of MenB vaccines for gonorrhoea prevention, independent of MenB IMD prevention, could also be considered for targeted vaccination of specific populations with high rates of acquiring gonococcal infection. Given its concentrated gonorrhoea epidemic, the UK has recently implemented a targeted 4CMenB vaccination programme, focusing on a relatively small subset of sexual health clinic attendees with the highest risk for acquisition, primarily gay, bisexual and other MSM with a bacterial STI diagnosis in the past year or multiple recent sex partners [60]. In addition to the results of current 4CMenB RCTs, real‐world follow‐up of the UK programme will be valuable for understanding the feasibility, uptake and potential impact of targeted MenB vaccination programmes for gonorrhoea prevention [60].

In countries with a history of MenB endemic disease or outbreaks, extending the benefits of MenB vaccines beyond meningococcal disease prevention to include protection against gonococcal infection could influence policy decisions related to MenB OMV vaccine introduction and use. The use of 4CMenB remains limited in many parts of the world. The first global policy guidance on MenB vaccines, through a WHO Strategic Advisory Group of Experts on Immunization (SAGE) position paper, is expected in 2026. WHO and partners are also working to understand the contexts in which meningococcal vaccines, including MenB‐containing vaccines, are being implemented as part of national immunization programmes. South Africa is an example of a country with a high general population prevalence of gonococcal infection and known low‐level endemic MenB disease, suggesting an efficacious vaccine might provide some benefits for both conditions. In South America, in the context of rising MenB disease rates, Chile and, more recently, Uruguay have introduced MenB vaccination programmes for infants, while Argentina and Brazil are currently discussing the potential inclusion of MenB vaccination in their national immunization programmes [68, 69]. Added benefits for gonorrhoea might alter decision‐making. Much of the current discussions relate to cost‐effectiveness, given the costs of the vaccine along with relatively low IMD incidence in most settings. Further cost‐effectiveness modelling is needed, incorporating both conditions, to understand how potential benefits of MenB vaccines for gonococcal infection prevention, in addition to their benefits for meningococcal disease, might tip the balance towards introduction in different settings, including LMICs.

In countries where the epidemiology of MenB IMD and gonococcal infection overlaps, or where 4CMenB is already in use, a key issue in expanding vaccine use for gonorrhoea is defining the target populations. The WHO gonococcal vaccine PPCs identify young people aged 10–24 years and populations at higher risk of acquiring gonococcal infection, such as MSM and female sex workers, as priority groups [20]. In contrast, because MenB IMD incidence is highest for infants and young children, most MenB vaccination programmes focus on infancy. However, overlap in epidemiology among adolescents and young adults in some settings presents an opportunity. Where MenB immunization programmes already include adolescents, as in parts of Australia, extending vaccination to also prevent gonococcal infection would be relatively straightforward, and could strengthen recommendations for both conditions. A key challenge is ensuring sufficient duration of protection against gonorrhoea, which peaks in most settings in the 20‐ to 24‐year‐old age group. A Phase IV evaluation of a state‐funded, school‐based adolescent 4CMenB vaccination programme in South Australia (vaccination at ages 15–16 years) found 42% vaccine effectiveness against gonococcal infection within 5 years of vaccination, with no apparent effectiveness beyond 5 years [70]. These findings suggest booster doses may be needed to extend protection into early adulthood. In settings such as the United States, where MenB vaccination is recommended for adolescents and young adults with frequent close contact to others, such as universities or the military, the alignment between MenB and gonorrhoea target age groups may be even greater.

Although vaccines specifically formulated to optimize efficacy against gonorrhoea remain an important goal, such vaccines may not be available for many years [8], and MenB vaccines could provide an earlier intervention for gonococcal control. The WHO gonococcal vaccine PPCs also highlight additional product and programmatic considerations for using MenB vaccines to prevent gonorrhoea, in advance of gonococcal‐specific vaccines [20]. For example, because they are already indicated for MenB disease, MenB vaccines may be acceptable with lower efficacy than would be required for a standalone gonococcal vaccine [20]. The level of observed efficacy in different population groups may influence whether manufacturers pursue a formal regulatory indication for gonorrhoea prevention or whether off‐label use of MenB vaccines would need to be considered, as was done in the UK [60]. Vaccines for meningitis may also be perceived as less stigmatizing than dedicated gonococcal vaccines that clearly target an STI. This may be an important factor for acceptability in an era of rising vaccine hesitancy, particularly for adolescent use. Vaccine acceptance may vary by target population; available data suggest gonococcal vaccines would be highly acceptable among MSM [71]. Costs and feasibility of delivering both types of vaccines outside of school‐based adolescent platforms will be critical issues, particularly in LMICs. Expansion of HIV prevention programmes and other sexual and reproductive health services might offer novel opportunities to deliver focused gonococcal vaccination.

Insights from the 4CMenB RCTs, including immunologic mechanisms of partial protection, efficacy in those with and without prior infection, and sex‐specific differences, can guide purposeful design and use of optimized gonococcal‐specific vaccines. These data can also guide next‐generation *Neisseria* vaccines. For example, a pentavalent MenABCWY vaccine (PENMENVY, GSK) has now been approved in the United States for use in 10‐ to 25‐year‐olds [72, 73]. Confirmed cross‐protection against gonorrhoea by 4CMenB would be anticipated to extend to this vaccine, which combines 4CMenB with MenACWY CRM197. Pentavalent vaccines could potentially broaden relevance to additional countries with both high gonorrhoea prevalence and IMD due to other meningococcal serogroups. Real‐world data from countries implementing MenB OMV vaccines in both targeted and universal vaccination programmes can further inform considerations pertaining to acceptability, feasibility and optimal delivery strategies, both for the use of MenB vaccines in gonorrhoea prevention as well as for future gonococcal vaccines [20, 60, 70].

## 5 Conclusions

Developing and evaluating vaccines to prevent gonococcal infection are an important pursuit for sexual and reproductive health, particularly due to rising gonococcal AMR. Observational data suggest OMV‐based vaccines designed to prevent MenB disease could provide cross‐protection against gonorrhoea. Results from five RCTs of MenB vaccines for the prevention of gonococcal infection are expected in 2026. If ongoing RCTs confirm MenB vaccine efficacy against gonorrhoea, it will be important to understand how these vaccines might be used across both conditions. We found data lacking in much of the world and little overlap in the epidemiology of gonococcal infection and meningococcal disease, both between and within countries. Nonetheless, several patterns emerged that provide insight into future use of MenB OMV vaccines for gonorrhoea. In some settings, introduction of MenB OMV vaccines could be considered for gonococcal infection alone—targeting high‐risk sub‐populations or, in countries with high general‐population prevalence, broadly targeting adolescents and young adults. Certain countries might have benefits for both conditions, which could tip the balance in terms of public health need and cost‐effectiveness in favour of implementation. Better delineating the public health value of MenB vaccines in terms of both pathogens is critical. This includes collecting better epidemiologic data on both conditions; improving understanding of disease outcomes related to gonorrhoea and potential effects of increasing AMR; and modelling the impact and cost‐effectiveness of vaccination for both conditions. Engaging with communities to better understand their preferences and needs, particularly among adolescents, young adults and high‐risk populations, and advanced planning for programmatic rollout and communication strategies will also be critical. RCTs of MenB vaccines for gonorrhoea and evaluation of real‐world programmes in the UK and Australia will not only inform future use of MenB vaccines, but also the development and use of more efficacious gonococcal‐specific vaccines [20, 62], a key priority for global sexual and reproductive health.

## Author Contributions

SLG and NB conceptualized the review and study approach. SLG, JR and NB finalized the methodology, with technical input from DAC, AD and M‐PP. SLG, JR, NB and DAC conducted the review and extracted relevant data. SLG and JR had direct access to and verified all underlying data reported in the manuscript. SLG and NB developed the first draft of the manuscript, and all authors participated in reviewing, providing technical input and editing manuscript drafts. All authors approved the final manuscript.

## Funding

The authors have nothing to report.

## Conflicts of Interest

DAC has received personal fees for scientific presentations for Sanofi Pasteur. RB reports contract research on behalf of the UK Health Security Agency for GSK, Pfizer and Sanofi Pasteur. HSM is an investigator on vaccine trials sponsored by industry; her institution receives funding from GSK, Pfizer and Sanofi Pasteur for investigator‐led research: she receives no personal payments from industry. MAS reports research grants and consultancy fees from GSK, Pfizer and Sanofi Pasteur. All other authors report no potential conflicts.

## Maps

The boundaries and designations used on the maps in this article do not imply the expression of any opinion whatsoever on the part of the World Health Organization concerning the legal status of any country, territory, city or area or of its authorities, or concerning the delimitation of its frontiers or boundaries. Dotted and dashed lines on maps represent approximate border lines for which there may not yet be full agreement.

## Supporting information




**Supporting File**: List of countries where Bexsero is registered and/or commercialized.

## Data Availability

No new data were generated for this article. All data used are from previously published or publicly available sources, which are referenced in the article.
