# Supplementary material for: Potential Use of Neisseria meningitidis Serogroup B Vaccines to Prevent Neisseria gonorrhoeae Infection: Epidemiological Considerations
Source: J Int AIDS Soc. 2026 Jul 24;29(Suppl 2):e70155. doi: 10.1002/jia2.70155 (PMC13400992; doi:10.1002/jia2.70155)
Supplement: Supplementary file 1 — Supporting File: List of countries where Bexsero is registered and/or commercialized. [file JIA2-29-e70155-s001.pdf]

GSK

Approval Cover Sheet for Data on File Summary

DoF Summary	
Drug/Compound #	BEXSERO
Author of DoF Summary	Nicolas Jamet
Date	January 15, 2026
DoF/Study Report Title	List of countries where Bexsero is registered and/or commercialized
Vault Doc Number (MI use only)	REF-309490

INITIAL APPROVAL

I confirm that the DoF Summary (attached) is accurate, has been reviewed and approved.
This information is appropriate for dissemination outside the company.

Nicolas Jamet	*Electronically signed by: Nicolas Jamet Reason: I am signing for the reasons as stated in the document.<br>Date: January 15, 2026 15:45 GMT+2*
Print name	Signature of Head of Data Generating Department Or Delegate
Director, Global Pricing & Market Access, Neisseria	15-Jan-26
Title	Date of Approval

Prior approval of abstract, poster, or platform presentations for presentation at congress as documented in Datavision

Name of Approving Manager: ____

Approvals for Revalidation/Revision of Existing DOF Summary (Required for Non-Clinical and Health Outcomes Data)

Print name	Signature of Head of Data Generating Department Or Delegate
Title	Date of Approval
Print name	Signature of Head of Data Generating Department Or Delegate
Title	Date of Approval
Print name	Signature of Head of Data Generating Department Or Delegate
Title	Date of Approval

Country	Actual Submission (MAA)	Approval	License Holder	Age Indication	Expiry date	Marketed
1. Argentina	13-Dec-2013	30-Sep-2015	GlaxoSmithKline Argentina S.A.	from 2 Months of age and older	30-Sep-2025	Yes
2. Australia	07-Oct-2011	14-Aug-2013	GSK Australia Pty Ltd	from 2 Months of age and older	Perpetual	Yes
3. Austria	21-Dec-2010	14-Jan-2013	GSK Vaccines S.r.l. Via Fiorentina 1 Siena IT	from 2 Months of age and older	Perpetual	Yes
4. Belgium	21-Dec-2010	14-Jan-2013	GSK Vaccines S.r.l. Via Fiorentina 1 Siena IT	from 2 Months of age and older	Perpetual	Yes
5. Brazil	15-Aug-2011	09-May-2016	GlaxoSmithKline Brasil Limitada	from 2 months to 50 years of age	09-May-2026	Yes
6. Bulgaria	21-Dec-2010	14-Jan-2013	GSK Vaccines S.r.l. Via Fiorentina 1 Siena IT	from 2 months of age and older	Perpetual	Yes
7. Canada	26-May-2011	06-Dec-2013	GlaxoSmithKline Inc.	from 2 months through 25 years of age	Perpetual	Yes
8. Chile	29-nov-2013	27 May 2014	GlaxoSmithKline Chile Farmaceutica Limitada	from 2 Months of age and older	27-May-2029	Yes
9. Colombia	15-Sept-2021	04-April-2024	GlaxoSmithKline Biologicals s.a. Rue de l'Institut 89 Rixensart BE	from 2 Months of age and older	Perpetual	Yes
10. Costa Rica	24-Nov-2023	ongoing	GSK Vaccines S.r.l. Via Fiorentina 1 Siena IT	from 2 months of age and older		No
11. Croatia	21-Dec-2010	14-Jan-2013	GSK Vaccines S.r.l. Via Fiorentina 1 Siena IT	from 2 Months of age and older	Perpetual	Yes
12. Cyprus	21-Dec-2010	14-Jan-2013	GSK Vaccines S.r.l. Via Fiorentina 1 Siena IT	from 2 Months of age and older	Perpetual	Yes
13. Czech Republic	21-Dec-2010	14-Jan-2013	GSK Vaccines S.r.l. Via Fiorentina 1 Siena IT	from 2 Months of age and older	Perpetual	Yes
14. Denmark	21-Dec-2010	14-Jan-2013	GSK Vaccines S.r.l. Via Fiorentina 1 Siena IT	from 2 Months of age and older	Perpetual	Yes
15. Dominican Republic	21-Nov-2023	13 March 2024	GSK Vaccines S.r.l. Via Fiorentina 1 Siena IT	from 2 months of age and older	14-Feb-2029	No
16. Egypt	25 June 2023	22 Feb 2024	GlaxoSmithKline S.A.E. - Cairo	from 2 months of age and older	21-Feb-2029	Yes

17. El Salvador	19-Feb-2024	29-Jul-2024	GSK Vaccines S.r.l. Via Fiorentina 1 Siena IT	from 2 months of age and older	25-Jul-2029	Planned
18. Estonia	21-Dec-2010	14-Jan-2013	GSK Vaccines S.r.l. Via Fiorentina 1 Siena IT	from 2 Months of age and older	Perpetual	No
19. Finland	21-Dec-2010	14-Jan-2013	GSK Vaccines S.r.l. Via Fiorentina 1 Siena IT	from 2 Months of age and older	Perpetual	Yes
20. France	21-Dec-2010	14-Jan-2013	GSK Vaccines S.r.l. Via Fiorentina 1 Siena IT	from 2 Months of age and older	Perpetual	Yes
21. Germany	21-Dec-2010	14-Jan-2013	GSK Vaccines S.r.l. Via Fiorentina 1 Siena IT	from 2 Months of age and older	Perpetual	Yes
22. Greece	21-Dec-2010	14-Jan-2013	GSK Vaccines S.r.l. Via Fiorentina 1 Siena IT	from 2 Months of age and older	Perpetual	Yes
23. Guatemala	21-Dec-2023	ongoing	GSK Vaccines S.r.l. Via Fiorentina 1 Siena IT	from 2 months of age and older		No
24. Hong Kong	13-Dec-2018	25-Jun-2019	GlaxoSmithKline Limited (Hong Kong)	from 2 Months of age and older	24-June-2029	Yes
25. Hungary	21-Dec-2010	14-Jan-2013	GSK Vaccines S.r.l. Via Fiorentina 1 Siena IT	from 2 Months of age and older	Perpetual	Yes
26. Iceland	21-Dec-2010	14-Jan-2013	GSK Vaccines S.r.l. Via Fiorentina 1 Siena IT	from 2 Months of age and older	Perpetual	No
27. Indonesia	19-May-2025	ongoing	GSK Vaccines S.r.l. Via Fiorentina 1 Siena IT	from 2 months of age and older		No
28. Ireland	21-Dec-2010	14-Jan-2013	GSK Vaccines S.r.l. Via Fiorentina 1 Siena IT	from 2 Months of age and older	Perpetual	Yes
29. Israel	06-Jan-2015	08-Mar-2016	GlaxoSmithKline (Israel) Ltd	from 2 Months of age and older	Perpetual	Yes
30. Italy	21-Dec-2010	14-Jan-2013	GSK Vaccines S.r.l. Via Fiorentina 1 Siena IT	from 2 Months of age and older	Perpetual	Yes
31. Latvia	21-Dec-2010	14-Jan-2013	GSK Vaccines S.r.l. Via Fiorentina 1 Siena IT	from 2 Months of age and older	Perpetual	No
32. Lichtenstein	21-Dec-2010	14-Jan-2013	GSK Vaccines S.r.l. Via Fiorentina 1 Siena IT	from 2 Months of age and older	Perpetual	Yes
33. Lithuania	21-Dec-2010	14-Jan-2013	GSK Vaccines S.r.l. Via Fiorentina 1 Siena IT	from 2 Months of age and older	Perpetual	Yes
34. Luxembourg	21-Dec-2010	14-Jan-2013	GSK Vaccines S.r.l. Via Fiorentina 1 Siena IT	from 2 Months of age and older	Perpetual	Yes

35.	Malaysia	31-May-2024	ongoing	GlaxoSmithKline Pharmaceutical Sdn Bhd Malaysia	from 2 Months of age and older		No
36.	Malta	21-Dec-2010	14-Jan-2013	GSK Vaccines S.r.l. Via Fiorentina 1 Siena IT	from 2 Months of age and older	Perpetual	Yes
37.	Mexico	31-Oct-2022	14-Apr-2025	GlaxoSmithKline Biologicals SA, Rue de Institut 89, Rixensart, 1330, Belgium	from 2 Months of age and older	14-Apr-2030	No
38.	Morocco	13-Jan-2025	ongoing	GlaxoSmithKline Maroc SA	from 2 months of age and older		No
39.	Netherlands	21-Dec-2010	14-Jan-2013	GSK Vaccines S.r.l. Via Fiorentina 1 Siena IT	from 2 Months of age and older	Perpetual	Yes
40.	New Zealand	16-Nov-2017	19-Jul-2018	GlaxoSmithKline (NZ) Ltd	from 2 Months of age and older	Perpetual	Yes
41.	Norway	21-Dec-2010	14-Jan-2013	GSK Vaccines S.r.l. Via Fiorentina 1 Siena IT	from 2 Months of age and older	Perpetual	Yes
42.	Panama	25-Jan-2024	27-Feb-2025	GSK Vaccines S.r.l. Via Fiorentina 1 Siena IT	from 2 Months of age and older	27 Feb 2030	No
43.	Philippines	29-Nov-2022	21-May-2024	GlaxoSmithKline Philippines, Inc	from 2 Months of age and older	21-May-2029	Yes
44.	Poland	21-Dec-2010	14-Jan-2013	GSK Vaccines S.r.l. Via Fiorentina 1 Siena IT	from 2 Months of age and older	Perpetual	Yes
45.	Portugal	21-Dec-2010	14-Jan-2013	GSK Vaccines S.r.l. Via Fiorentina 1 Siena IT	from 2 Months of age and older	Perpetual	Yes
46.	Romania	21-Dec-2010	14-Jan-2013	GSK Vaccines S.r.l. Via Fiorentina 1 Siena IT	from 2 Months of age and older	Perpetual	Yes
47.	Russian Federation	31-Aug-2020	20 April 2022	JSC GlaxoSmithKline Trading, Russia	from 2 Months of age and older	20 Apr 2027	No
48.	Saudi Arabia	03-Oct-2019	19-Aug-2020	Glaxo Saudi Arabia Ltd. Jeddah, KSA	from 2 Months of age and older	19-Aug-2025	Yes
49.	Slovakia	21-Dec-2010	14-Jan-2013	GSK Vaccines S.r.l. Via Fiorentina 1 Siena IT	from 2 Months of age and older	Perpetual	Yes
50.	Slovenia	21-Dec-2010	14-Jan-2013	GSK Vaccines S.r.l. Via Fiorentina 1 Siena IT	from 2 Months of age and older	Perpetual	Yes
51.	South Africa	05-Dec-2019	04-Jul-2023	GlaxoSmithKline South Africa (Pty) Ltd 39 Hawkins Avenue, Epping Industria 1, 7460, South Africa	from 2 Months of age and older	04-Jul-2028	No

52. South Korea	26-Nov-2020	19-May-2022	GlaxoSmithKline Korea Limited - Seoul	from 2 Months of age and older	18 May 2033	Yes
53. Spain	21-Dec-2010	14-Jan-2013	GSK Vaccines S.r.l. Via Fiorentina 1 Siena IT	from 2 Months of age and older	Perpetual	Yes
54. Sweden	21-Dec-2010	14-Jan-2013	GSK Vaccines S.r.l. Via Fiorentina 1 Siena IT	from 2 Months of age and older	Perpetual	Yes
55. Switzerland	11-Dec-2014	23-Nov-2017	GlaxoSmithKline AG	from 2 months to 24 years of age	Perpetual	Yes
56. Taiwan	27-Dec-2019	19-May-2021	GlaxoSmithKline FAR EAST B.V.-TAIPEI	from 2 Months of age and older	19-May-2026	Yes
57. Thailand	30-Aug-2022	25-Jul-2024	GlaxoSmithKline (Thailand) Ltd. - Bangkok	from 2 Months of age and older	24-Jul-2031	Yes
58. Turkey	26-Dec-2014	06-Sep-2018	GlaxoSmithKline ILACLARI SAN. VE TIC. A.S	from 2 Months of age and older	Perpetual	Yes
59. Ukraine	15-Nov-21	07-Oct-2022	GSK Export Limited UK	from 2 Months of age and older	07-Oct-2027	Yes
60. U.S.A.I	24-Jul-2014	23-Jan-2015	GlaxoSmithKline Biologicals s.a. Rue de l'Institut 89 Rixensart BE	from 10 to 25 years of age	Perpetual	Yes
61. United Arab Emirates	04-Oct-2020	21-Dec-2020	GSK Vaccines S.r.l. Via Fiorentina 1 Siena IT	from 2 months of age and older	20-Dec-2025	Yes
62. United Kingdom and Northern Ireland	21-Dec-2010	14-Jan-2013	GlaxoSmithKline UK Limited<br>980 Great West Road<br>Brentford<br>Middlesex<br>TW8 9GS<br>United Kingdom	from 2 Months of age and older	Perpetual	Yes
63. Uruguay	23-Dec-2013	02-Oct-2014	GSK Vaccines S.r.l. Via Fiorentina 1 Siena IT	from 2 Months of age and older	02-Oct-2029	Yes
64. Vietnam	31 Dec 2020	19 May 2023	GSK Pharma Viet Nam Company Limited – Ho Chi Mich City	from 2 months of age and older	19 May 2026	Yes

Bexsero Immunization Programs:

- Infants: 20 countries with either National or Regional Immunization Programs
  - National: Andorra; Chile; Czechia; France; Germany; Greece; Ireland; Italy; Lichtenstein; Lithuania; Luxembourg; Malta; New Zealand; Portugal; San Marino; Spain; Switzerland; UK; Uruguay
  - Regional: Australia (South Australia; Tasmania; Queensland; Northern Territory)
- Adolescents: 6 countries with either National or Regional IP:
  - National: Czechia; Switzerland; Lichtenstein; United States (Shared-Clinical Decision Making)
  - Regional: Italy (13/20 Regions); Australia (same as for infants). France has no formal recommendation, but reimbursement for eligible population;

Countries with programs focused on at risk individuals (e.g. Canada; or NZ) were excluded.
